# Interface Engineering Modulated Valley Polarization in MoS_2_/*h*BN Heterostructure

**DOI:** 10.3390/nano13050861

**Published:** 2023-02-25

**Authors:** Fang Li, Hui Zhang, You Li, Yibin Zhao, Mingyan Liu, Yunwei Yang, Jiamin Yao, Shaolong Min, Erjun Kan, Yi Wan

**Affiliations:** 1 MIIT Key Laboratory of Semiconductor Microstructure and Quantum Sensing, Department of Applied Physics, Nanjing University of Science and Technology, Nanjing 210094, China; 2 Institute of Physics and Electronic Information, Yunnan Normal University, Kunming 650500, China

**Keywords:** molybdenum disulfide, hexagonal boron nitride, valley polarization, photoluminescence quantum yield, relaxation time

## Abstract

Layered transition metal dichalcogenides (TMDs) provide a favorable research platform for the advancement of spintronics and valleytronics because of their unique spin-valley coupling effect, which is attributed to the absence of inversion symmetry coupled with the presence of time-reversal symmetry. To maneuver the valley pseudospin efficiently is of great importance for the fabrication of conceptual devices in microelectronics. Here, we propose a straightforward way to modulate valley pseudospin with interface engineering. An underlying negative correlation between the quantum yield of photoluminescence and the degree of valley polarization was discovered. Enhanced luminous intensities were observed in the MoS_2_/*h*BN heterostructure but with a low value of valley polarization, which was in stark contrast to those observed in the MoS_2_/SiO_2_ heterostructure. Based on the steady-state and time-resolved optical measurements, we reveal the correlation between exciton lifetime, luminous efficiency, and valley polarization. Our results emphasize the significance of interface engineering for tailoring valley pseudospin in two-dimensional systems and probably advance the progression of the conceptual devices based on TMDs in spintronics and valleytronics.

## 1. Introduction

Two-dimensional (2D) polarized materials, including ferromagnets [1], ferroelectrics [2], and ferrovalley materials [3,4], demonstrate peculiar behaviors at the quantum realm. Valley pseudospin, which represents the energy band extremes in momentum space, normally exists in periodic solid materials [5,6]. The addressability of valley pseudospin enables the utilization of the momentum states of carriers as a brand-new paradigm in data coding and information handling. Using the research strategies of spintronics [7,8] for reference, a similar concept, valleytronics, arose naturally and vigorously [9]. The novel scientific connotation associated with the manipulation of the valley degree of freedom may result in a transformative impact. Referring to theoretical predictions about the intrinsic properties closely related to the valley pseudospin, rapid experimental advances [10,11,12,13,14] have been performed to observe and manipulate the valley polarization in a way similar to real spin.

Recently, the successful isolation and further experimental characterizations of 2D materials, including but not limited to graphene, hexagonal boron nitride (*h*BN), and TMDs, enriched our cognition of valley physics [15]. The spatial symmetry breaking along with the time-reversal symmetry enable two sets of the individually addressable valleys, K and K’ points in the first Brillouin zone, for TMDs [16]. Especially when the layered TMDs, such as MoS_2_, WS_2_, and MoSe_2_, are mechanically exfoliated from bulk-phase crystals and thinned down to monolayers, a marvelous transition in their electronic structure occurs, viz the evolution from an indirect bandgap to a direct one. The direct bandgaps of monolayer TMDs normally lie in the near-infrared and visible spectral ranges of approximately 1~2 eV, suitable for the investigation of valley pseudospin-related optoelectronic applications. Van der Waals (vdW) heterostructures composed of 2D materials offer a fascinating research platform for tailoring artificial composite constructions with unique properties, novel phenomena [17,18], and widespread potential applications [19,20,21]. The concept of engineering material properties via fabricating mixed-dimensional vdW heterostructures can also be employed to manipulate valley polarization with great convenience and low cost for spintronics and valleytronics [22,23].

Here, we performed circularly polarization-dependent photoluminescence (PL) measurements upon MoS_2_ monolayers transferred atop *h*BN nanoflakes and SiO_2_/Si wafers at room temperature, prepared with the chemical vapor deposition (CVD) method. Substantial variations were observed in these two vertically stacked systems, MoS_2_/*h*BN and MoS_2_/SiO_2_, especially in their luminous performance. An underlying opposite relationship between the intensity of luminescence and the degree of valley polarization was observed. Specifically, a stronger exciton luminescence was observed in the MoS_2_/*h*BN heterostructure but with a lower degree of valley-polarized emission, while a weaker luminous performance and a higher degree of valley polarization were obtained in the MoS_2_/SiO_2_ heterostructure. We infer that there exists a connection to be examined between the luminous efficiency and the valley polarization. According to the time-resolved circularly polarization-dependent PL measurements, a lower degree of valley polarization is observed in samples that exhibit longer exciton lifetimes. Our work reveals an inverse relationship between luminescence efficiency and the valley polarization value, and emphasizes the significance of interface engineering for modulating the intrinsic new degrees of freedom in ultrathin 2D polarized materials. It may potentially deepen our understanding of 2D quantum systems and advance the realization of the emerging practical applications for next-generation information storage and processing.

## 2. Materials and Methods

**Figure 1:**
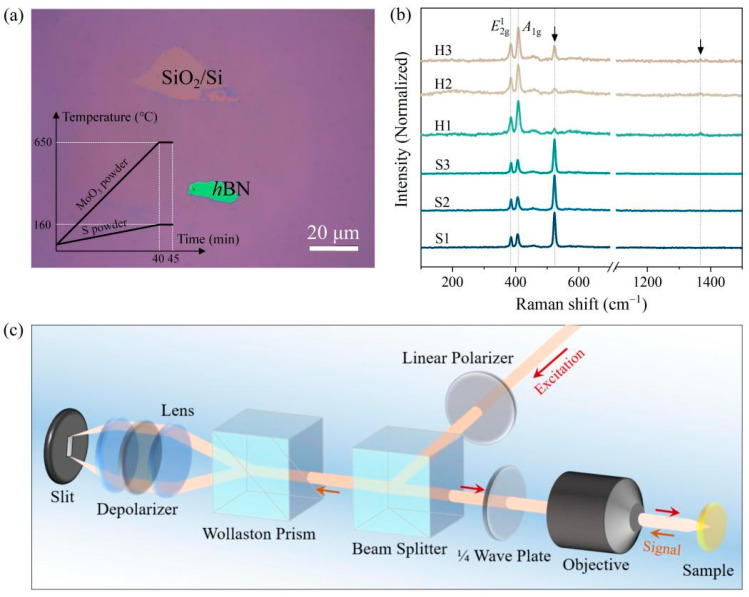
(**a**) Optical image of continuous MoS_2_ monolayer films prepared with the CVD method. There is a typical *h*BN flake obtained with mechanical exfoliation on the commonly used SiO_2_ (285 nm)/Si wafer before CVD growth. Inset: Temperature and time parameters for the CVD growth of the MoS_2_ monolayers. (**b**) Typical Raman spectra obtained from the MoS_2_ monolayer prepared atop *h*BN (H1, H2, and H3) and SiO_2_ (S1, S2, and S3). (**c**) The schematic diagram of the experimental setup for collecting the circularly polarized photoluminescence signals from the MoS_2_ sample.

Large-area, continuous MoS_2_ monolayers with a lateral size of several millimeters were routinely prepared with a standardized CVD process in a commercial dual-temperature-zone furnace [24,25] using high-purity S and MoO_3_ powders as the solid-state powder sources. Mechanically exfoliated *h*BN flakes were transferred atop SiO_2_/Si wafers with a 285 nm oxidation layer before CVD growth. Highly purified argon was employed as the transport gas. The growth temperature and the required time parameters we used for the CVD process are shown in the inset of Figure 1a. The programming temperatures for the two respective zones, in which the S and MoO_3_ powders were placed, were elevated from room temperature to 160 and 650 °C in 40 min and remained unchanged for 5 min. Once the reaction and deposition processes finished, it was cooled naturally. Generally speaking, the typical growth of MoS_2_ crystals atop SiO_2_/Si normally results in equilateral triangle shapes under thermodynamically stable conditions. As shown in the optical microscopy image, the MoS_2_ samples were successfully deposited onto the 285 nm SiO_2_/Si wafer with some mechanically exfoliated *h*BN flakes randomly distributed atop its surface (Figure 1a). It can be observed that numerous discrete MoS_2_ monolayers connected to each other and merged into a continuous single-layer film. The continuous single-layer film typically exhibits a lateral dimension up to several hundreds of micrometers, beneficial for probing many discrete locations within a sample via an optical means. Yu et al. previously reported that, unlike the growth of MoS_2_ on SiO_2_ substrates, the growth of MoS_2_ on *h*BN normally follows a lattice alignment epitaxial growth mode [26], where two structurally equivalent 0° and 60° stacked MoS_2_/*h*BN are presented.

Figure 1b presents the Raman scattering spectra acquired from six distinct locations, corresponding to the MoS_2_ samples prepared atop the *h*BN and SiO_2_ substrates, respectively. The characteristic feature located at approximately 520.7 cm^−1^ is attributed to the underneath Si substrate used for wavenumber calibration. Due to the reliability and repeatability of our synthetic strategy, we can observe two characteristic peaks of MoS_2_, *E*^1^_2g_ and *A*_1g_, as well as the signal from *h*BN (≈1366.4 cm^−1^), as shown in the Raman spectra labeled as H1–H3, confirming that MoS_2_ is successfully grown on the *h*BN flake. The peak interval between the two characteristic features of MoS_2_, *E*^1^_2g_ and *A*_1g_, can help us determine the number of layers of the MoS_2_ samples quantitatively and rapidly (Appendix A). One accessible mode for *h*BN is the in-plane *E*_2g_ mode located at approximately 1366.4 cm^−1^ (Appendix B).

The vdW heterostructures comprised of 2D materials serve as an inspiring platform for tailoring physicochemical properties, exploring novel quantum effects, and ultimately fabricating conceptually new devices [17,18]. A classical paradigm is the demonstration of the fascinating Hofstadter butterfly observed in artificial vertically stacked moiré superlattices comprised of graphene and *h*BN [27,28]. The strategy to tailor material properties with interface engineering is also available for the vertically stacked structure comprised of TMDs and *h*BN, which enables the exploration of valley polarization. The circular polarization-sensitive PL measurement was performed in a home-made micro-zone setup as displayed in Figure 1c. The linearly polarized excitation light is converted to the circularly polarized one by a broadband quarter-wave plate. A 50× objective (N.A. 0.55) is used to collect the emission signals from the MoS_2_ monolayers. The left-handed and right-handed circularly polarized light signals (*σ*+ and *σ*−) are converted to horizontal and vertical linearly polarized light signals (*I_σ_*_+→*σ*+_ and *I_σ_*_+→_*_σ_*_−_), respectively, via a broadband quarter-wave (1/4λ) plate. The two linearly polarized light beams can be separated in real space with a Wollaston prism and then focused to two spots positioned at the entrance slit of the spectrometer equipped with the charged coupled device (CCD) cooled at −75 °C. The intensities of these two orthogonally linearly polarized beams, corresponding to the *σ*+ and *σ*− components of the PL signal, are recorded simultaneously with the CCD. By doing so, we greatly reduce the error caused by laser power fluctuation. The steady-state circularly polarized PL spectra were captured under resonant excitation with a continuous-wave 633 nm laser. For the time-resolved PL measurements, a pulsed linearly polarized Ti:sapphire laser with the wavelength of 405 nm was employed.

## 3. Results and Discussion

### 3.1. Crystal Structure of MoS_2_ and the Coupled Valley-Spin Excitonic Transition Rules

**Figure 2:**
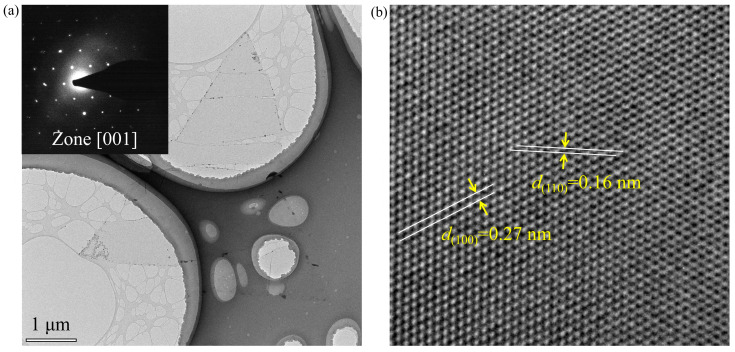
(**a**) TEM image of a MoS_2_ monolayer triangle transferred atop a carbon-film-coated copper microgrid. Inset: SAED patterns recorded along the zone [001] axis of MoS_2_. (**b**) Atomic-level resolution TEM image of MoS_2_. A periodic triangular packing arrangement of transition metal molybdenum atoms is clearly observed.

Transmission electron microscopy (TEM) provides a powerful tool for examining the morphology and lattice structure of low-dimensional materials. As shown in Figure 2a, a MoS_2_ monolayer triangle was transferred atop a carbon-film-coated TEM copper microgrid in the presence of a relatively complete geometric morphology. The recorded selected area electron diffraction (SAED) spots (inset, Figure 2a) exhibit a typical hexagonal pattern, consistent with that of MoS_2_. The lattice spacings of approximately 0.27 nm and 0.16 nm are clearly visible along the {100} and {110} planes of MoS_2_, respectively (Figure 2b). The atomic-level resolution TEM image along with the corresponding SAED patterns demonstrate that the CVD-synthesized MoS_2_ monolayer possesses excellent crystal quality with a hexagonal lattice structure.

**Figure 3:**
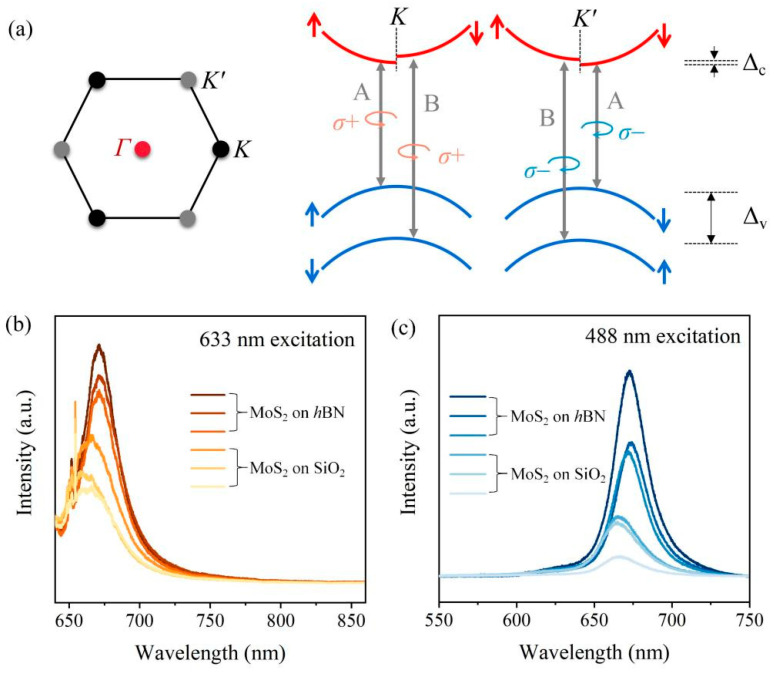
(**a**) Schematic illustration of the coupled valley-spin excitonic transition rules at the K and K’ valleys in momentum space, where Δ_c_(Δ_v_) denotes the amplitude of the energy splitting induced by spin-orbit coupling in the CBM and VBM in the MoS_2_ monolayer. (**b**,**c**) PL spectra obtained from the representative MoS_2_ samples on *h*BN and on SiO_2_. The excitation wavelengths are 633 nm (**b**) and 488 nm (**c**).

From the top view of the crystal structure in MoS_2_, a hexagonal honeycomb lattice structure can be observed that generates two sets of degenerate-but-not-equivalent valleys, K and K’, at the edges of the first Brillouin zone (left panel, Figure 3a). These valleys that are degenerate in energy exhibit a huge splitting in the valence band (Δ_v_ = ~148 meV) induced by spin-orbit coupling and a much smaller one in the conduction band (Δ_c_ = ~3 meV), which is also depicted for completeness (right panel, Figure 3a), for the MoS_2_ monolayer [29,30,31,32]. The K and K’ valleys are differentiated by the opposite spin orientations corresponding to the valence band maximum (VBM) and conduction band minimum (CBM). As schematically displayed in the right panel of Figure 3a, combined with the time reversal symmetry, the spin orientations of the K and K’ valleys are anti-symmetric, enabling a locking of the spin and the valley degree of freedom. The remarkable difference guarantees the valley-dependent optical selection rules that the direct excitonic transitions should obey, including A and B, in the MoS_2_ monolayer. To be specific, the circularly polarized lights with left-handed helicity (*σ*+) excite the excitonic transitions in the K valleys exclusively, whereas those lights with right-handed helicity (*σ*−) only couple to the K’ valleys. In our previous report [24], we reported that the A excitonic emission from the MoS_2_ monolayer on the *h*BN flake exhibited a ubiquitous enhancement compared with that on SiO_2_/Si. As plotted in Figure 3b,c, the intensities of the PL signals from MoS_2_ on *h*BN are much stronger than those obtained from MoS_2_ on SiO_2_, both under 633 nm and 488 nm excitation.

### 3.2. Steady-State Circularly Polarized PL Spectra

**Figure 4:**
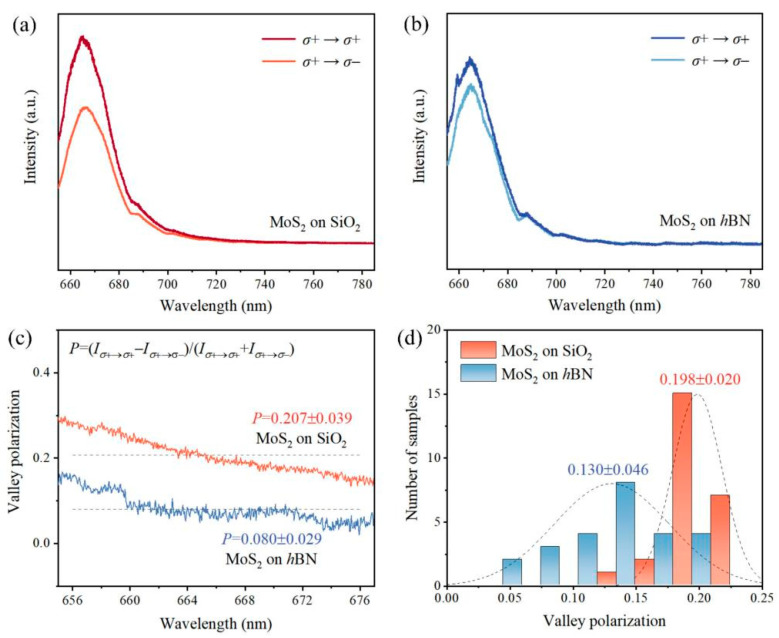
(**a**,**b**) Room-temperature circularly polarized PL spectra obtained from the MoS_2_ monolayers on SiO_2_ and *h*BN under a resonant continuous-wave excitation. (**c**) Degree of valley polarization determined with *P* = (*I_σ_*_+→*σ*+_−*I_σ_*_+→*σ*−_)/(*I_σ_*_+→*σ*+_+*I_σ_*_+→*σ*−_), where *I_σ_*_+→*σ*+_ and *I_σ_*_+→*σ*−_ represent the intensities of the left-handed and right-handed circularly polarized PL components, respectively. (**d**) Statistical histogram of the calculated valley polarization value for the MoS_2_ monolayers on SiO_2_ (orange) and on *h*BN (blue) summarized from 50 samples. The summarized valley polarization values are 0.198 for MoS_2_ on SiO_2_ and 0.130 for MoS_2_ on *h*BN.

The valley polarization-resolved luminous property appears a typical representative among the abundant unique physical properties for the MoS_2_ monolayer. The vertical stacking of MoS_2_ and *h*BN enables us to explore the valley polarization utilizing our home-made circular polarization PL measurement system. We further performed the PL measurements upon the MoS_2_ monolayer on *h*BN and SiO_2_ under resonant excitation with a 633 nm laser, widely employed in valley pseudospin-related luminous property investigation. The value for the degree of valley polarization obtained from the MoS_2_ monolayers with different substrates underneath was calculated based on the measured polarization-resolved PL spectra involving *σ*− and *σ*+ components. As shown in Figure 4a and b, we obtained the PL spectra by using the 1.96 eV (633 nm) laser radiation as excitation, that is with the left-handed helicity (*σ*+) on resonance with the A excitonic transition. We determined the degree of valley polarization quantitatively [10,11] as

(1) $$P=\frac{I_{\sigma +\to \sigma +}-I_{\sigma +\to \sigma -}}{I_{\sigma +\to \sigma +}+I_{\sigma +\to \sigma -}}$$

where *I_σ_*_+→*σ*+_ and *I_σ_*_+→*σ*−_ denote the intensities of the left-handed and right-handed circularly polarized PL signals, respectively, which are excited with a left-handed circular excitation laser. As plotted in Figure 4c, the average valley polarization values, ranging from 656 nm to 676 nm, were calculated to be 0.207 for MoS_2_ on SiO_2_ and 0.080 for MoS_2_ on *h*BN. It can be observed, among the wavelength range of the direct A excitonic emission for the MoS_2_ monolayer, the valley polarization for MoS_2_ on *h*BN is always lower than that observed from the MoS_2_ monolayers on SiO_2_.

Based on the numerical analyses of 25 MoS_2_ monolayer samples on *h*BN, the statistical average for the degree of valley polarization is *P* = 0.130 ± 0.046 (Figure 4d), which demonstrates a relatively low degree of valley polarization in the MoS_2_/*h*BN heterostructure at room temperature (300 K). In stark contrast to that, the degree of valley polarization for the MoS_2_ monolayer deposited atop the SiO_2_ wafer under the identical measurement conditions is relatively high. The statistical average value equals approximately 0.198 ± 0.020 from which an important message can be delivered that the interaction widely existent in 2D materials/supporting substrates may play a critical role in the modulation of valley pseudospin in 2D polarized materials. Combined with the measurement results mentioned above, an enhanced PL intensity and a reduced valley polarization are observed in the MoS_2_/*h*BN heterostructure. Why does there exist a noticeably opposite relationship between luminous intensity and valley polarization? To answer this question and further clarify the underlying mechanism, we further performed the time-resolved circularly polarization-dependent PL measurements at room temperature.

For the steady-state conditions excited with a continuous wave (CW) laser, the degree of valley polarization, *P*, can be determined with

(2) $$P=\frac{P_{0}}{1+\frac{2\tau_{e}}{\tau_{v}}}$$

under a rate model, where *P*_0_ denotes the initial polarization, and *τ_e_* and *τ_v_* represent the exciton and valley relaxation times, respectively. The derivative process is provided in Appendix C. The value of *P* increases with either an increase in valley lifetime *τ_v_* or a decrease in exciton lifetime *τ_e_*, as clearly observed from Equation (2). Previous reports did not identify the influence of substrates on the valley relaxation time *τ_v_* for MoS_2_, among which the measured values are close numerically and even the supporting substrates are different. All the as-synthesized MoS_2_ monolayers shown in Figure 4 were excited with exactly the same excitation wavelength, pumping power, and exposure time, and exposed to nearly the identical environment. Thus, it is assumed that the valley relaxation time *τ_v_* and the initial polarization *P*_0_ are the same. It can be inferred that the degree of valley polarization *P* will decrease if the exciton relaxation time increases.

### 3.3. Time-Resolved Circularly Polarized PL Spectra

According to the theory in semiconductor physics, the exciton relaxation time *τ_e_* is closely related to both radiative and non-radiative recombination times through the following relational expression:

(3) $$\frac{1}{\tau_{e}}=\frac{1}{\tau_{r}}+\frac{1}{\tau_{nr}}$$

The time parameters, *τ_r_* and *τ_nr_*, represent the radiative and non-radiative lifetimes, respectively. For transition metal dichalcogenides, the non-radiative lifetimes can be orders of magnitude shorter than the radiative recombination times. In other words, the magnitude relationship *τ_nr_* << *τ_r_* exists in MoS_2_ monolayers. Hence, there exists an approximation relation *τ_e_* ≈ *τ_nr_*. For a certain material, the radiative recombination time *τ_r_* can be regarded as a constant. Since the luminous intensity is in direct proportion to quantum yield (*QY*),

(4) $$QY=\frac{1}{1+\frac{\tau_{r}}{\tau_{nr}}}\approx \frac{1}{1+\frac{\tau_{r}}{\tau_{e}}}$$

If the radiative recombination time remains unchanged, a longer exciton relaxation time will lead to a higher *QY* and, thus, an enhanced PL intensity.

**Figure 5:**
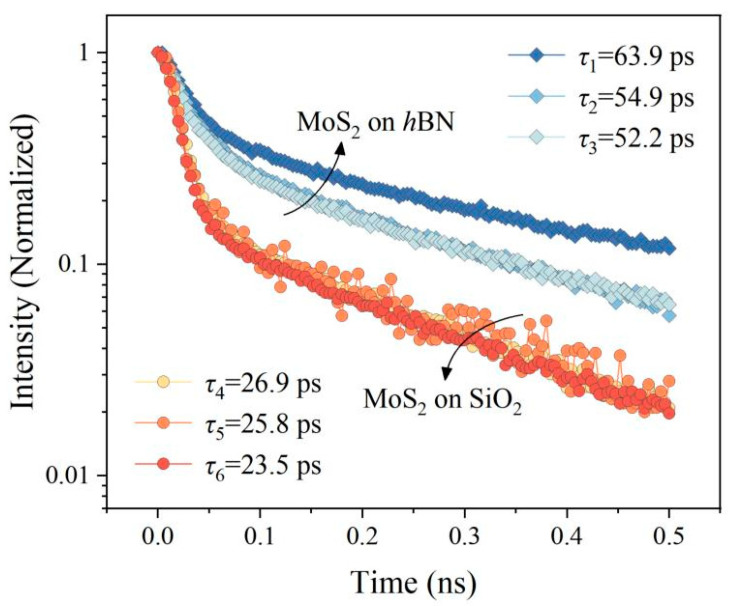
The circularly polarized time-resolved PL spectra obtained from the MoS_2_ monolayers on *h*BN and SiO_2_, all of which were measured at room temperature. By the deconvolution fitting, the exciton lifetimes could be obtained.

We further performed time-resolved photoluminescence (TRPL) measurements to examine the exciton dynamics and capture the corresponding fluorescence lifetime with which our hypothesis mentioned above may be verified. The measured TRPL spectra are plotted in Figure 5. Two systems, including the MoS_2_ monolayers deposited atop *h*BN and SiO_2_, were measured, which behave remarkably different from each other in the intensity of luminescence and the degree of valley polarization. A pulsed, linearly polarized Ti:sapphire laser with the wavelength of 405 nm was employed to pump the direct excitons into the two degenerate-but-not-equivalent valleys, K and K’, in the MoS_2_ monolayer simultaneously via an optical means. The pulsed optical pumps were realized with an optical parametric amplification. The subsequent luminescent signals created from the K and K’ valleys could then be collected and directed to a time-resolved CCD detector. The dynamic experimental results were quite similar to those observed in the steady-state PL measurement results.

As mentioned above, the MoS_2_ monolayers on *h*BN and SiO_2_ display noticeably different PL intensity as well as distinguishing valley polarization behaviors. The time-resolved emission spectra measured from these samples, as presented here, also exhibit significantly different exciton attenuation kinetics. MoS_2_ on *h*BN possesses a longer exciton lifetime than MoS_2_ on SiO_2_. Compared with SiO_2_, *h*BN is chemically inert and has no defect states or hanging bonds on its surface. This may result in less disorder to the MoS_2_ monolayer [33], which is mainly stemmed from extrinsic effects, for example, defects and trap states. The difference in the exciton relaxation time explains the enhanced PL intensity and lower valley polarization of MoS_2_ on *h*BN.

## 4. Conclusions

In conclusion, we successfully synthesized a MoS_2_ monolayer on *h*BN nanoflakes via the CVD method and performed room-temperature circularly polarized PL measurements upon the MoS_2_ samples with different substrates underneath. We observed that there exists an apparent inverse correlation between the intensity of PL signals and the value of valley polarization, which originates from the variations in exciton relaxation time. Compared to MoS_2_ monolayers atop SiO_2_, the MoS_2_ monolayers atop *h*BN exhibit a relatively low degree of valley polarization. An enhanced PL intensity and a longer exciton lifetime were also experimentally consolidated in the MoS_2_/*h*BN system. Our discovery suggests a pathway to tailor carrier dynamics via crystal modification, which can be realized by introducing an appropriate amount of defects or non-radiative recombination sites. By doing so, the room-temperature valley polarization can be effectively modulated, and the coupling between degenerate valleys can be attenuated, which provides new strategies for the realization of state-of-the-art devices in spintronics and valleytronics.

## Figures and Tables

**Figure A1:**
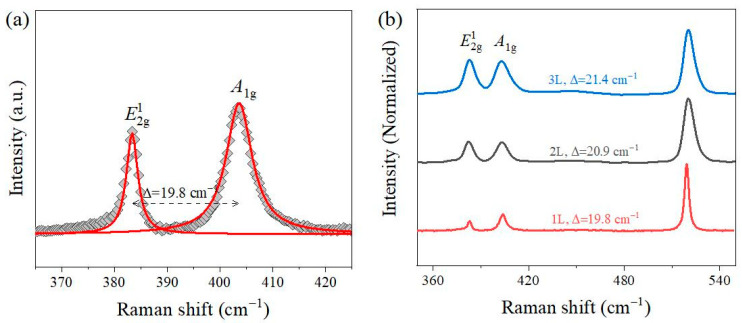
(**a**) Zoom-in Raman spectrum of 1L MoS_2_. (**b**) Typical Raman spectra obtained from 1L, 2L, and 3L MoS_2_ prepared atop SiO_2_. The Raman peak located at approximately 520.7 cm^−1^ corresponding to Si is used for wavenumber calibration and intensity normalization.

**Figure A2:**
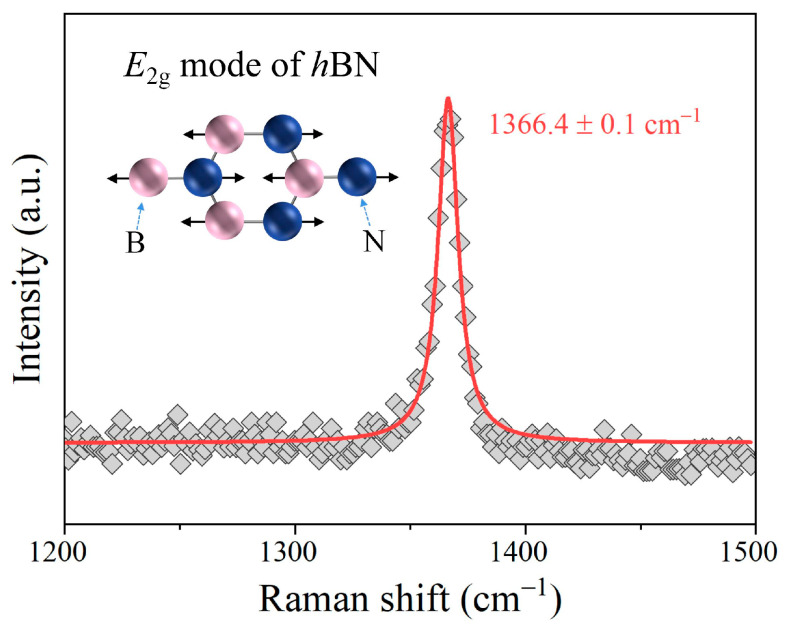
Raman peak located at approximately 1366.4 cm^−1^ corresponding to *h*BN. The left top schematic image indicates the Raman-active mode *E*_2g_ of *h*BN.

## Data Availability

All data concerning this study are contained in the present manuscript and in previous articles, whose references have been provided.

## Appendix A

Li et al. proposed a general thickness identification technique for atomic-scale, few-layer MoS_2_ on dielectric substrates [34], such as SiO_2_. The peak interval between the two characteristic features of MoS_2_, *E*^1^_2g_ and *A*_1g_, can determine the number of layers of the MoS_2_ samples quantitatively and rapidly.

The zoom-in Raman spectrum of the MoS_2_ monolayer on SiO_2_ is provided in Figure A1a. We can clearly observe the characteristic modes *E*^1^_2g_ and *A*_1g_ of MoS_2_, the peak interval (~19.8 cm^−1^) between which is consistent with the criterion for CVD-synthesized MoS_2_ monolayers on SiO_2_. Figure A1b plots the Raman spectra obtained from 1L, 2L, and 3L MoS_2_ on SiO_2_, the thickness of which was determined with the atomic force microscope technique. The three characteristic peaks located among 360~540 cm^−1^ correspond to two characteristic peaks of MoS_2_, *E*^1^_2g_ and *A*_1g_, as well as the signal from the underneath Si substrate (520.7 cm^−1^). In the series of Raman spectra, corresponding to 1L, 2L, and 3L MoS_2_, there exists a monotonous variation trend in the peak interval between *E*^1^_2g_ and *A*_1g_.

The detailed values of peak positions, as shown in Figure A1b, are summarized in Table A1. With the increasing number of layers, there exists a decrease in the peak position of *E*^1^_2g_ mode, an increase in the peak position of *A*_1g_ mode, and an increase in the peak interval Δ, consistent with the experimental results previously reported by Li et al. [34]. As mentioned above, it is a feasible calibration method to determine the number of layers of the few-layer MoS_2_ samples based on the amplitude of the peak interval Δ.

**Table A1:** The Raman spectral peak positions of few-layer MoS_2_.

Number of Layer	*E*^1^_2g_ (cm^−1^)	*A*_1g_ (cm^−1^)	Δ (cm^−1^)
1	383.4	403.2	19.8
2	382.3	403.2	20.9
3	382.1	403.5	21.4

## Appendix B

Raman scattering spectroscopy provides a non-destructive technique for studying *h*BN. One accessible mode is the in-plane *E*_2g_ mode at approximately 1366.4 cm^−1^ [35]. As displayed in Figure A2, a narrow feature is a representative of highly ordered, defect-free *h*BN. As we can observe from Figure 1b, the two characteristic peaks of MoS_2_, *E*^1^_2g_ and *A*_1g_, as well as the signal from *h*BN (≈1366.4 cm^−1^) obtained from the samples labeled as H1 to H3 in the main text, indicate that MoS_2_ is successfully synthesized atop the mechanically exfoliated *h*BN flakes.

## Appendix C

Under a rate model [10], the rate equation for the neutral exciton populations at the two degenerate-but-not-equivalent K and K’ valleys can be mathematically described as follows:

(A1) $$\frac{dn_{K}}{dt}=g_{K}-\frac{n_{K}}{\tau_{nr}}-\frac{n_{K}}{\tau_{exciton\to trion}}-\frac{n_{K}-n_{K^{\prime }}}{\tau_{v}}$$

(A2) $$\frac{dn_{K^{\prime }}}{dt}=g_{K^{\prime }}-\frac{n_{K^{\prime }}}{\tau_{nr}}-\frac{n_{K^{\prime }}}{\tau_{exciton\to trion}}+\frac{n_{K}-n_{K^{\prime }}}{\tau_{v}}$$

where *g_K_* and *g_K_*_’_ represent the excitation rate optically pumped by left-handed helicity (*σ*+) coupled to the excitonic transitions in the K valleys and right-handed helicity (*σ*−) coupled to the K’ valleys, respectively. Besides, *τ_nr_*, *τ_exciton__→__trion_*, and *τ_v_* denote the non-radiative relaxation time, the scattering relaxation time from neutral exciton to charged tightly bound trion [36,37], and the intervalley relaxation time between K and K’ valleys, respectively. In fact, under a relatively high measuring temperature [36] or a relatively low carrier injection concentration [37], as in our experiments, the scattering process from exciton to trion can be ignored, which means *τ_exciton__→__trion_* can be viewed as an infinite.

Under the steady-state excitation for the neutral exciton (A) generated in the K valley, as employed in our experiment, the right-handed helicity excitation is zero, viz *g_K_*_’_ = 0, and the material system is in a state of dynamic equilibrium, which indicates that

(A3) $$\frac{dn_{K}}{dt}=\frac{dn_{K^{\prime }}}{dt}=0$$

We determine the degree of valley polarization quantitatively as

(A4) $$P=\frac{I_{\sigma +\to \sigma +}-I_{\sigma +\to \sigma -}}{I_{\sigma +\to \sigma +}+I_{\sigma +\to \sigma -}}$$

based on the polarization-dependent PL intensities. The valley polarization can also be written as

(A5) $$P=\frac{n_{K}-n_{K^{\prime }}}{n_{K}+n_{K^{\prime }}}$$

based on the polarization-dependent PL intensities. Thus, for the steady-state conditions excited with a CW laser, the degree of valley polarization, *P*, can be determined with

(A6) $$P=\frac{1}{1+\frac{2\tau_{nr}}{\tau_{v}}}$$

According to the theory in semiconductor physics, the exciton relaxation time *τ_e_* is closely related to both radiative and non-radiative recombination times through the following relational expression, *τ_e_*^−1^ = *τ_r_*^−1^ + *τ_nr_*^−1^, in which the time parameters, *τ_r_* and *τ_nr_*, represent the radiative and non-radiative lifetimes, respectively. For transition metal dichalcogenides, the non-radiative lifetimes can be orders of magnitude shorter than radiative recombination times, viz *τ_nr_* << *τ_r_*. Hence, there exists an approximation relation *τ_e_* ≈ *τ_nr_*. Considering the derivation in initial polarization that is closely related to some parameters, including but not limited to excitation laser wavelength, exposure time, and possible intervalley generation, we multiply the numerator with a coefficient *P*_0_ additively:

(A7) $$P=\frac{P_{0}}{1+\frac{2\tau_{e}}{\tau_{v}}}$$

Consequently, the less-than-perfect (much lower than the ideal 100%) degree of valley polarization observed in the circularly optical measurement results is attributed to inevitable intervalley processes and the competition between the valley lifetime *τ_v_* and the recombination relaxation time *τ_e_*.
